# Frataxin deficiency induces lipid accumulation and affects thermogenesis in brown adipose tissue

**DOI:** 10.1038/s41419-020-2253-2

**Published:** 2020-01-23

**Authors:** Riccardo Turchi, Flavia Tortolici, Giulio Guidobaldi, Federico Iacovelli, Mattia Falconi, Stefano Rufini, Raffaella Faraonio, Viviana Casagrande, Massimo Federici, Lorenzo De Angelis, Simone Carotti, Maria Francesconi, Maria Zingariello, Sergio Morini, Roberta Bernardini, Maurizio Mattei, Piergiorgio La Rosa, Fiorella Piemonte, Daniele Lettieri-Barbato, Katia Aquilano

**Affiliations:** 1 Department Biology, University of Rome Tor Vergata, via della Ricerca Scientifica, Rome, Italy; 2 Department of Molecular Medicine and Medical Biotechnologies, University of Naples Federico II, Naples, Italy; 3 Department of Systems Medicine, University of Rome Tor Vergata, Rome, Italy; 4 Unit of Microscopic and Ultrastructural Anatomy, University Campus Bio-Medico, Rome, Italy; 5 Interdepartmental Service Center-Station for Animal Technology (STA), University of Rome Tor Vergata, Rome, Italy; 6 Unit of Neuromuscular and Neurodegenerative Diseases, IRCCS Bambino Gesù Children’s Hospital, Rome, Italy; 7 IRCCS Fondazione Santa Lucia, 00143 Rome, Italy

**Keywords:** Biological sciences, Biochemistry

## Abstract

Decreased expression of mitochondrial frataxin (FXN) causes Friedreich’s ataxia (FRDA), a neurodegenerative disease with type 2 diabetes (T2D) as severe comorbidity. Brown adipose tissue (BAT) is a mitochondria-enriched and anti-diabetic tissue that turns excess energy into heat to maintain metabolic homeostasis. Here we report that the FXN knock-in/knock-out (KIKO) mouse shows hyperlipidemia, reduced energy expenditure and insulin sensitivity, and elevated plasma leptin, recapitulating T2D-like signatures. FXN deficiency leads to disrupted mitochondrial ultrastructure and oxygen consumption as well as lipid accumulation in BAT. Transcriptomic data highlights cold intolerance in association with iron-mediated cell death (ferroptosis). Impaired PKA-mediated lipolysis and expression of genes controlling mitochondrial metabolism, lipid catabolism and adipogenesis were observed in BAT of KIKO mice as well as in FXN-deficient T37i brown and primary adipocytes. Significant susceptibility to ferroptosis was observed in adipocyte precursors that showed increased lipid peroxidation and decreased glutathione peroxidase 4. Collectively our data point to BAT dysfunction in FRDA and suggest BAT as promising therapeutic target to overcome T2D in FRDA.

## Introduction

Friedreich’s ataxia (FRDA) is an inherited autosomal recessive neurodegenerative disorder. It is caused by mutation in the gene encoding mitochondrial protein frataxin (FXN)^1^. The primary function of FXN is to direct the mitochondrial synthesis of iron–sulfur clusters (Fe/S), which are essential parts of several mitochondrial enzymes, including mitochondrial respiratory chain complexes and aconitase^2^.

Deficiency in mitochondrial respiration, mitochondrial iron accumulation and oxidative stress are claimed as the main pathogenic factors in FRDA. Mitochondrial dysfunction mostly affects heart^3^ and cerebellum at the level of dentate nucleus^4,5^. Ferroptosis is a form of iron-mediated and lipid-mediated programmed cell death recently implicated in neurodegenerative diseases including FRDA. Notably, increased susceptibility to ferroptosis was found in primary patient-derived fibroblasts and murine fibroblasts from a mouse FRDA model^6^.

FRDA is characterized by a variable phenotype. Besides neurological symptoms, cardiomyopathy^3^ and systemic metabolic alterations occur^7^, which can predispose to diabetes development and cause premature death. In particular, patients with FRDA experience a greater risk of abnormal glucose homeostasis, in the form of both insulin resistance and glucose intolerance^8^. Increased blood cholesterol and triglycerides levels have been observed in FRDA patients^9,10^. To date, the major cause of T2D occurrence in FRDA patients seems to be related to impairment of mitochondria that in pancreatic β-cells are fundamental in generating signals that trigger and amplify insulin secretion^11^.

Brown adipose tissue (BAT) is a high oxidative tissue extremely rich in mitochondria that highly expresses the thermogenic protein uncoupling protein 1 (UCP1). BAT has emerged as a key regulator of glucose, lipid, and insulin metabolism^12–14^. Actually, BAT activity requires the uptake of substrates from the circulation, mostly free fatty acids (FAs), but also glucose, successfully leading to hypolipidemic and hypoglycemic effects, which can significantly improve insulin sensitivity and exert a protective role in the pathogenesis of T2D^15^. FAs liberated from intracellular triglycerides through the action of lipases are also critical for BAT thermogenesis^16^. Interestingly, abnormal accumulation of intracellular lipids has been observed in patients’ cells as well as in animal models^10,17–19^, pointing to a possible inefficient lipolysis.

Importantly, it has been discovered that decreased activity of BAT is associated with insulin resistance and T2D^20,21^. Despite this potential clinical importance, the regulation of BAT in FDRA patients is not well-investigated.

In this work we show that FXN deficiency significantly affects lipolysis as well as thermogenic and adipogenic cascade and increases susceptibility to ferroptosis in thermogenic adipocyte precursors. Overall these data suggest that BAT impairment could be at center stage of T2D development in FRDA patients.

## Results

### Alteration of basal metabolic parameters in KIKO mice

**Fig. 1:**
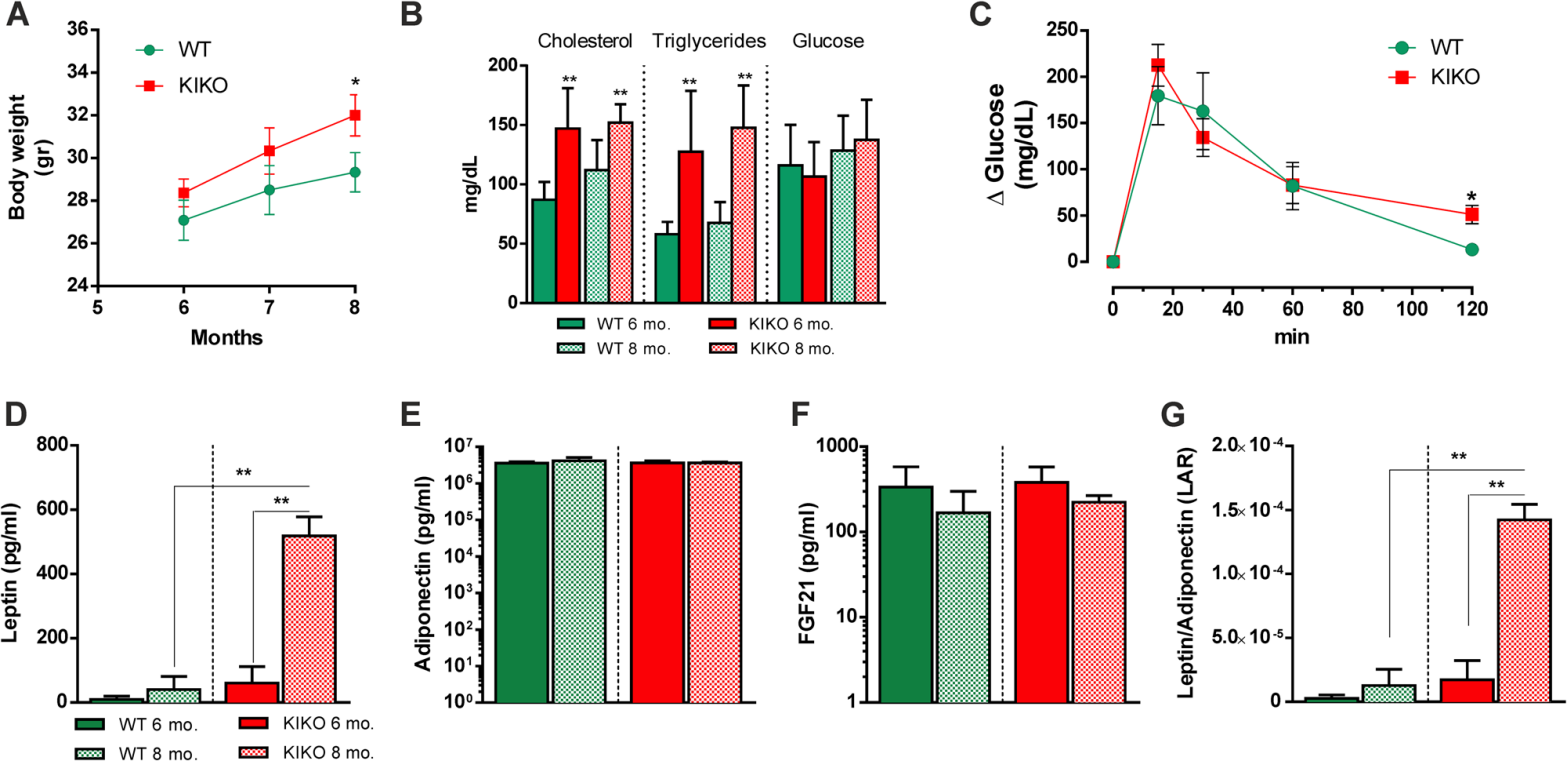
Bio-clinical and adipokine analyses revealed dyslipidemia and insulin resistance in KIKO mice. **a** Body weight recorded at different ages (*n* = 12 each group; *p < 0.05 vs. age-matched WT mice). **b** Bio-clinical analyses of fasting glycaemia and lipidemia (triglycerides and cholesterol) carried out at different ages (*n* = 12 each group; **p < 0.001 vs. age-matched WT mice). **c** Oral glucose tolerance test (OGTT) carried out in 8 months old mice (*n* = 6 each group; *p < 0.05 vs. WT mice). **d**–**g** Serum levels of leptin **d**, adiponectin **e**, FGF21 **f**, and leptin to adiponectin ratio **g** determined in mice at different ages (*n* = 6 each group; **p < 0.01).

Herein, we used KIKO mouse that represents a suitable in vivo model for studying neurodegeneration as well as metabolic complications in FRDA^17,22^. We firstly monitored body weight and a trend to gain weight was observed in 6 months KIKO mice that becomes significant at 8 months of age (Fig. 1a). In contrast, food and water intake were never found changed (data not shown). We have performed bio-clinical analyses and, among the tested parameters, a significant increase of triglycerides and cholesterol levels was detected in KIKO mice both at 6 and 8 months of age (Fig. 1b). Other metabolic parameters such as fasting glycaemia (Fig. 1b) as well as markers of general organ functions (total plasma proteins), including kidney (creatinine, urea) and liver (albumin) resulted unaltered (data not shown), suggesting that hyperlipidemia represents an early event in FRDA. Even though fasting glycaemia appeared unaffected, oral glucose tolerance test (OGTT) carried out in 8-month-old KIKO mice revealed that glycaemia remained higher than WT mice at 120 min from glucose administration (Fig. 1c).

The circulating levels of adipose tissue-secreted cytokines, i.e. adipokines, nicely reflect body metabolic state and are considered valid markers to monitor insulin resistance^23,24^. Through Luminex® Multiplex Assay, we found that KIKO mice underwent a prominent raise of plasma leptin levels at 8 months of age (Fig. 1d), while other adipokines, such as adiponectin and FGF21 remained unchanged (Fig. 1e, f). The increase in leptin, and more specifically the leptin to adiponectin ratio (LAR), is considered a useful tool to evaluate insulin resistance^25^. Accordingly, 8 months old KIKO mice showed increased LAR compared to WT mice (Fig. 1g).

**Fig. 2:**
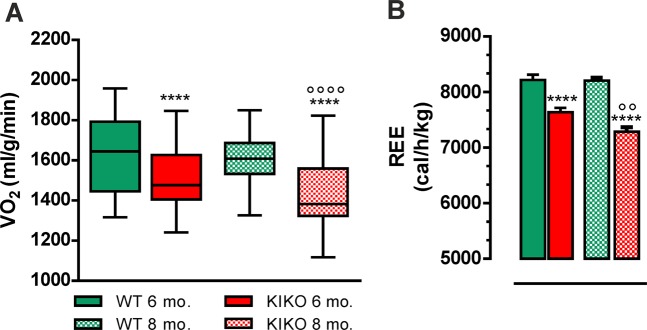
Indirect calorimetry indicated decreased oxygen consumption and energy expenditure in KIKO mice. **a, b** Oxygen consumption (VO_2_) **a** and resting energy expenditure (REE) **b** measured by indirect calorimetry at different ages (*n* = 12; each group; ****p < 0.0001 vs. age-matched WT mice, °°°°*p* < 0.0001, °°*p* < 0.01 vs. 6 months KIKO mice).

We have then characterized metabolic profile of KIKO mice by performing indirect calorimetry. Before measurements, all mice were acclimatized for 48 h into individual metabolic chambers at 25 °C, with free access to food (standard diet) and water. The respiratory exchange ratio (RER), which is expressed as VCO_2_/VO_2_, was similar in WT and KIKO mice and around 1.0 indicating carbohydrates as the predominant fuel source (data not shown). At 6 months, the recorded oxygen consumption (VO_2_) was lower in KIKO than WT mice, with KIKO mice showing a further decrease of VO_2_ at 8 months (Fig. 2a). Moreover, KIKO mice showed a decrease of resting energy expenditure (REE) at 6 months (Fig. 2b). This parameter was altered at higher extent at 8 months of age pointing to a progressive dysfunction in basal BAT thermogenic capacity (Fig. 2b).

### KIKO mice show altered mitochondrial function and lipid accumulation in BAT

**Fig. 3:**
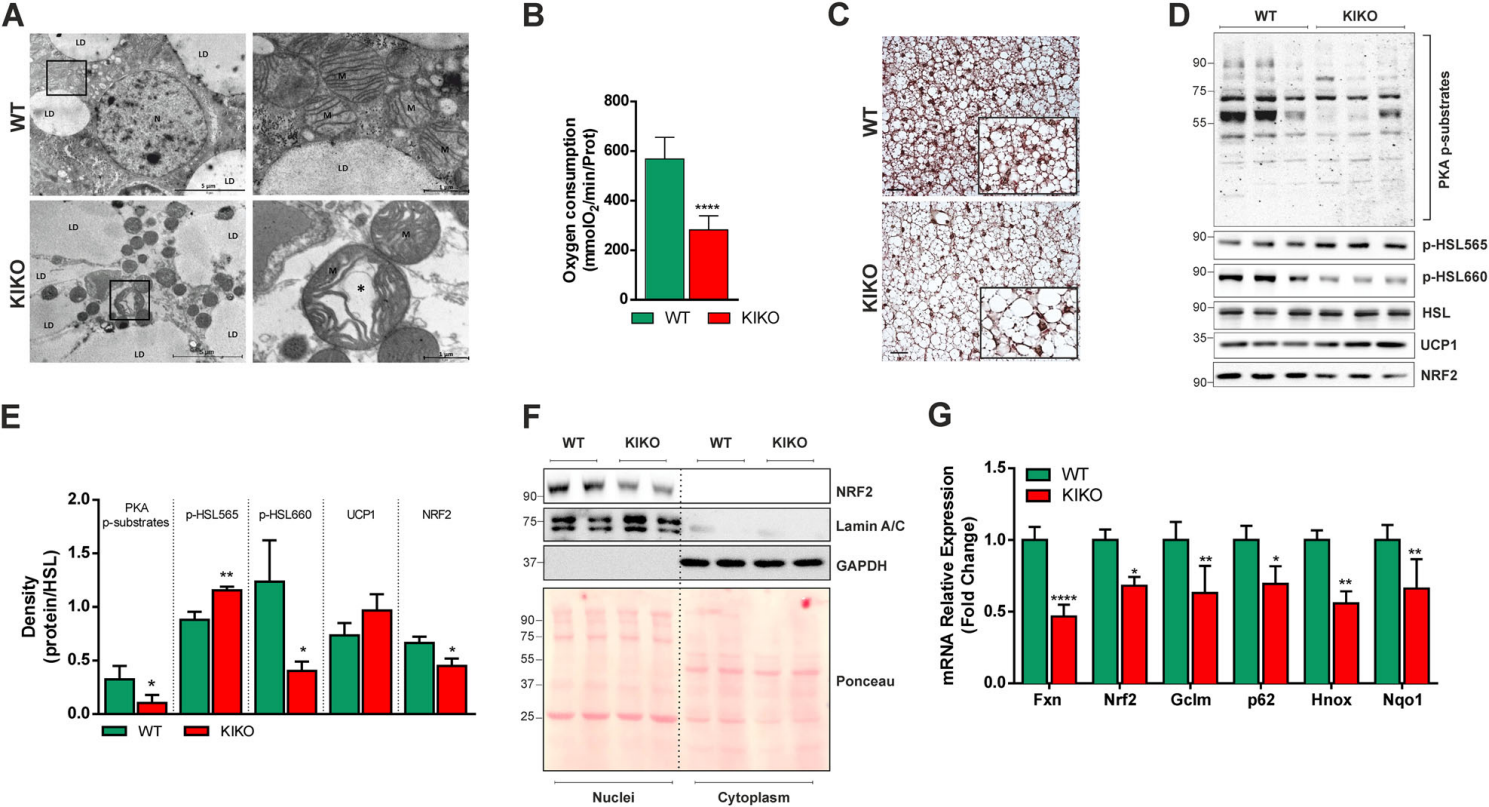
Mitochondria of KIKO mice show altered mitochondrial morphology and respiratory function. **a** Representative transmission electron microscopy pictures of BAT from 6-month-old WT and KIKO mice are reported in *left panels* (×5800). Images with high power field (×18,500) are reported in *right panels*. A representative mitochondrion of KIKO mice with altered ultrastructure was evidenced with an asterisks (*). LD lipid droplets; N nucleus; M mitochondria. **b** Oxygen consumption determined through a polarographic method on crude mitochondria isolated from BAT of 6 months old mice (*n* = 6 each group; ****p < 0.0001 vs. WT). **c** Representative BAT histology images after staining with H&E (*n* = 3 each group). Scale bars, 25 µm. **d, e** Representative immunoblots **d** of total HSL, phospho-active (p-HSL660) and phospho-inactive HSL (p-HSL565), PKA phosphorylated substrates (PKA p-substrates), UCP1 and NRF2 in total BAT homogenates, and densitometric analyses of the immunoreactive bands **e**. HSL was used as loading control (*n* = 6 each group, *p < 0.05, **p < 0.01 vs. WT). **f** Western blot analysis of NRF2 in nuclear and cytoplasmic fraction of BAT (*n* = 2 each group). Lamin A/C and GAPDH were used as loading control for nuclear and cytoplasmic fraction, respectively. **g** RT-qPCR analysis of Fxn, Nrf2, and its down-stream genes in BAT (*n* = 6; *p < 0.05, **p < 0.01, ***p < 0.001 vs. WT).

BAT activity strongly depends on mitochondrial lipid oxidation to produce heat^14^. As dysfunctional FXN affects mitochondrial oxidative capacity, we supposed that BAT oxidative metabolism could be altered. This prompted us at firstly analyzing BAT mitochondria at ultrastructural level through transmission electron microscopy. Typical mitochondria (abundant, large, and rich in cristae) were present in BAT of WT mice (Fig. 3a). On the contrary, in KIKO mice the mitochondria appeared lower in number and enlarged with disorganized and thickened cristae (Fig. 3a). We then compared the basal oxygen consumption in mitochondria isolated from BAT and, as expected, the oxygen consumption was lower in KIKO than WT mice (Fig. 3b).

Accumulation of lipid droplets (LDs) and increased lipogenesis have been previously described in fibroblasts of FRDA patients and cardiomyocytes of KIKO mice^17^. In line with these data, we found that BAT of KIKO mice has higher LD dimension with respect to WT mice (Fig. 3c). We have tested whether this event was dependent on defective lipolysis that importantly contributes to LDs turnover. BAT lipolysis strongly relies on cAMP-dependent protein kinase A (PKA) that coordinates lipolytic machinery activation through hormone/phospho-dependent mechanisms. To this end, we evaluated the level of the PKA-phosphorylated substrates as well as the lipases directly responsible for LDs degradation. As reported in the immunoblot in Fig. 3d, PKA substrates were reduced in their phosphorylated levels, suggesting a general inhibition of PKA activity. Among the PKA substrates the hormone-sensitive lipase (HSL) is included, as it is activated via the phosphorylation of Ser660. While the basal levels of HSL remained unaltered, the phospho-active levels of HSL were lower in KIKO compared to WT mice (Fig. 3d, e). The levels of phosphorylation at Ser565 (phospho-inactive form) were instead increased, indicating an impaired basal HSL activity. However, the levels of UCP1 protein did not undergo significant changes with respect to WT mice (Fig. 3d, e). According to what reported in other studies on mouse model and patients^26,27^, we found a significant decrease of the transcription factor nuclear factor, erythroid 2 like 2 (NRF2 or NFE2L2) (Fig. 3d, e) that, besides being an up-stream modulator of the expression of antioxidant genes and protecting against oxidative stress and ferroptosis^28^, positively regulates enzymes involved in mitochondrial FA oxidation^29^. In order to analyze the activity of NRF2, we performed nuclei isolation in BAT explants. As reported in Fig. 3f, the protein content of NRF2 was decreased in nuclei collected from BAT of KIKO mice compared to WT mice. The RT-qPCR analysis confirmed the downregulation of Fxn mRNA and revealed that the expression of Nrf2 as well as of its well-known downstream genes (i.e. Gclm, p62, Hmox, Nqo1) was significantly affected (Fig. 3g), confirming the reduction of NRF2 activity.

### KIKO mice display affected expression of genes related to lipid utilization and thermogenesis

**Fig. 4:**
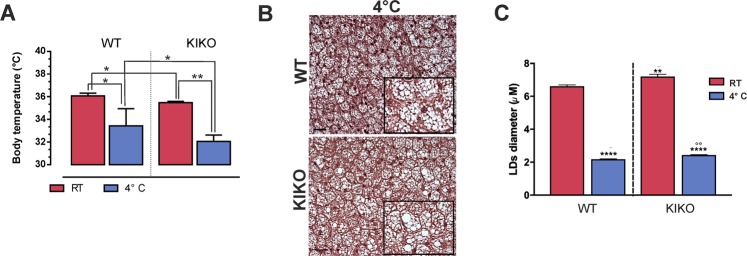
KIKO mice have reduced cold tolerance. **a** Body temperature measured in 6-month-old mice after 12 h cold exposure (*n* = 6 each group; *p < 0.05, **p < 0.01). **b** Representative histology images of BAT from mice exposed to cold after staining with H&E (*n* = 3 each group). Scale bars, 25 μm. **c** Lipid droplets (LDs) diameter measurement in three fields for each BAT histology through ImageJ (300 droplets total at least for each sample). BAT of WT and KIKO mice at RT (see **c**) or exposed to cold **b** for 12 h were analyzed (*n* = 3, ****p < 0.0001 vs. RT, **p < 0.001 vs. WT mice at RT, °°*p* < 0.001 vs. WT mice at 4 °C).

As we observed an alteration of mitochondrial respiration and lipid catabolism in BAT of KIKO mice, we hypothesized that BAT thermoregulatory activity could be compromised. To test this, we exposed mice to cool temperature (4 °C) for 12 h and a lower body temperature was measured in KIKO mice both at room temperature (RT, 25 °C) as well as at 4 °C (Fig. 4a). Remarkably, histochemical analysis of cold-exposed BAT showed that KIKO mice maintained a higher LD size with respect to WT mice (Fig. 4b). In particular, the measurement of LD size at RT confirmed their higher dimension in KIKO mice compared to WT mice (Fig. 4c). Cold exposure promoted a marked reduction of LDs diameter both in WT and KIKO mice; however, in cold-exposed BAT of KIKO mice LDs were larger than WT mice (Fig. 4c).

**Fig. 5:**
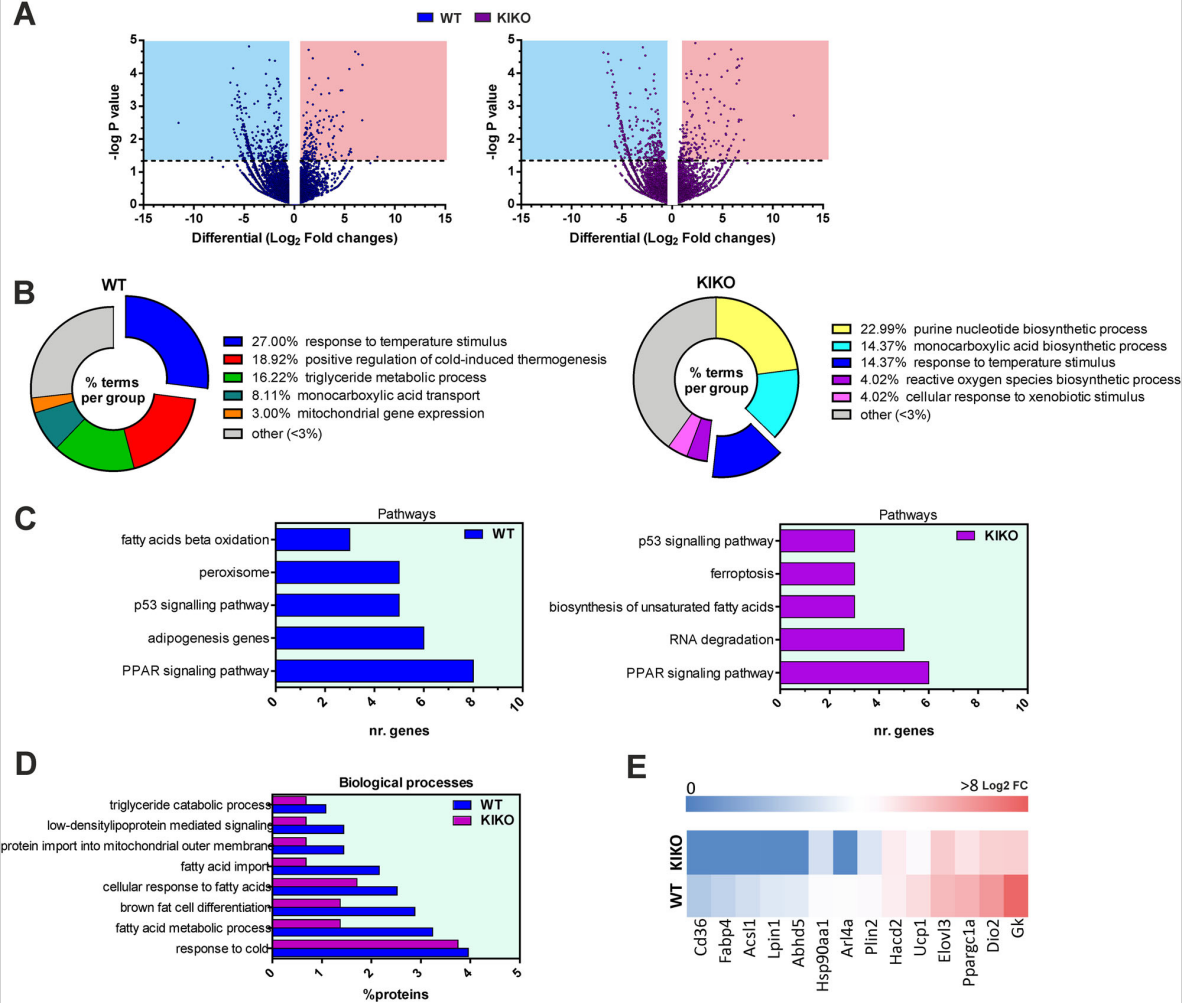
RNAseq analysis revealed reduced expression of genes related to BAT activity in KIKO mice. **a** Volcano plot representation of differential gene expression analysis in BAT of 6-month-old WT (*upper panel*) and KIKO mice (*lower panel*) exposed to cold for 12 h. Light blue and red squares mark the genes with significantly decreased or increased expression, respectively (*p* < 0.05, *n* = 3). **b**, **c** Biological processes **b**, KEGG and Wiki pathways **c** significantly up-regulated in BAT of WT (*left panels*) and KIKO mice (*right panels*) upon cold exposure determined through ClueGo plugin of Cytoscape v3.7 platform (Benjamini-corrected *p-*values < 0.05). **d** Comparative analysis of significantly up-regulated biological processes (*p* < 0.05) in WT mice and KIKO mice determined through Funrich v3.0. **e** Heatmap showing representative genes significantly up-regulated (*p* < 0.05) in WT mice compared to KIKO mice.

To more deeply decipher the cause(s) of cold intolerance, we performed BAT transcriptome profiling using an ultra-deep unbiased RNA sequencing (RNA-seq) approach. The RNA samples analyzed were prepared from BAT of WT and KIKO mice maintained at RT or exposed to cold for 12 h. A summary of the results obtained through RNAseq is reported in the volcano plots depicted in Fig. 5a. Through pair-wise differential gene expression, we found that about 200 genes were up-regulated (Log_2_FC > 0.58; *p* < 0.05) upon cold exposure both in WT and KIKO mice. In order to determine which biological processes or pathways were overexpressed, the list of up-regulated genes was used as the input for a functional enrichment analysis. By using the plugin ClueGo in the Cytoskape 3.7.1 platform, as expected we found that the response to temperature stimulus was the biological process significantly up-regulated either in WT or KIKO mice exposed to cold with respect to their controls, even though KIKO mice showed a lower enrichment with respect to WT mice (Fig. 5b). The biological processes found overrepresented in WT mice were: (1) positive regulation of cold-induced thermogenesis, (2) triglyceride metabolic process (including lipolytic genes), (3) monocarboxylic acid transport (including fatty acid transporters), and (4) mitochondrial gene expression (Fig. 5b, *left panel*), in line with the notion that thermogenesis is accompanied by FA transport and degradation, and expression of mitochondrial proteins. Interestingly, genes mapping to categories pertaining purine nucleotide and monocarboxylic acid biosynthetic processes (including FAs biosynthesis genes) were found overexpressed in KIKO mice exposed to cold compared to KIKO mice at RT (Fig. 5b, *right panel*). Moreover, in cold-exposed KIKO mice we found an enrichment of reactive oxygen species (ROS) biosynthetic process and cellular response to xenobiotic stimulus (Fig. 5b, *right panel*).

The analysis of KEGG and Wiki pathways showed that genes belonging to PPAR signaling, notably induced during thermogenesis, were significantly overrepresented both in cold-exposed WT and KIKO mice; however, the number of genes found in this pathway was lower in KIKO than WT mice (Fig. 5c). The same trend was observed for the p53-signaling pathway. Among the top enriched pathways, WT mice also had adipogenesis, peroxisome, and beta-oxidation (Fig. 5c*, left panel*); by contrast, RNA degradation, biosynthesis of FAs and ferroptosis were among the enriched pathways found in BAT of cold-exposed KIKO mice (Fig. 5c, *right panel*). Ferroptosis is a more recently recognized cell death caused by iron overload^30,31^, suggesting that in KIKO mice the thermogenic response of BAT is blunted and likely accompanied by iron/ROS-induced cell death.

To complement our analysis, we alternatively examined transcriptomic data through Funrich v3.0 and found that the response to cold was the biological process significantly and similarly enriched in WT and KIKO mice upon cold exposure (Fig. 5d). Brown fat cell differentiation and other biological processes related to lipid metabolism were found overrepresented both in WT and KIKO mice. However, in KIKO mice a lower percentage of genes pertaining to these processes was found compared to WT mice (Fig. 5d). In Fig. 5e, a heatmap including some representative genes strictly involved in adipocyte differentiation, heat production, and thermogenic-related metabolic re-adaptation is illustrated. The heatmap shows that KIKO mice undergo a lower up-regulation of these genes compared to WT mice, arguing that cold intolerance of KIKO mice could be ascribed to defective activation of adipocytes differentiation, thermogenic pathway, impaired BAT lipid catabolism and/or oxidative capacity.

**Fig. 6:**
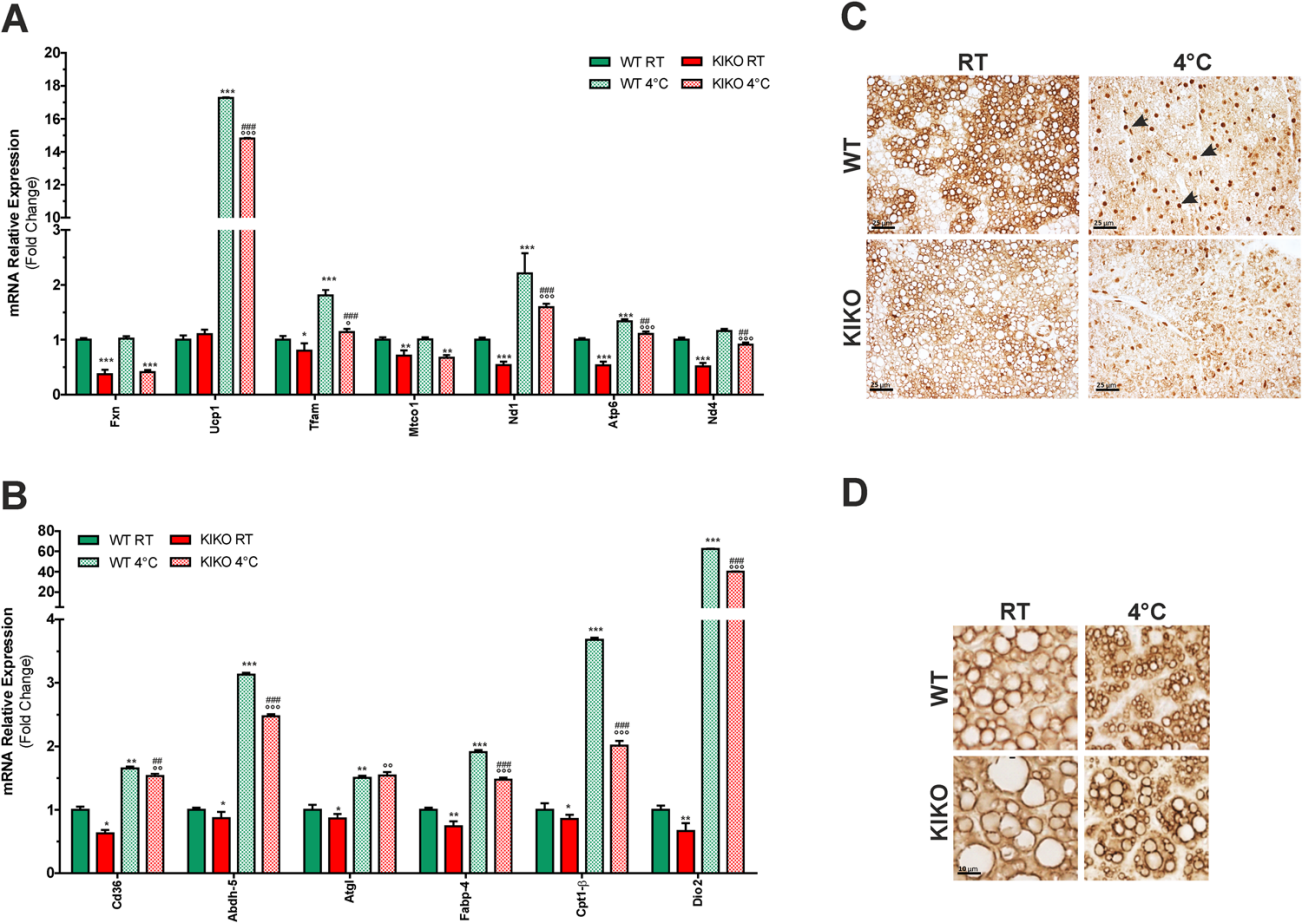
The expression of genes related to mitochondrial respiration, thermogenesis, and lipolytic pathways are altered in BAT of KIKO mice. **a**, **b** RT-qPCR analysis of mitochondrial genes **a** and genes related to lipid metabolism **b** in BAT of 6-month-old mice (*n* = 6; *p < 0.05, **p < 0.01, ***p < 0.001 vs. WT; °*p* < 0.05, °°*p* < 0.01, °°°*p* < 0.001 vs. KIKO; ^##^*p* < 0.01, ^###^*p* < 0.001 vs. WT at 4 °C). **c, d** Representative immunohistochemical analyses of phospho-PKA substrates **c** and perilipin-1 **d** (*n* = 3 mice each group). Black arrows **c** indicate some nuclei showing positivity to the immunostaining of phospho-PKA substrates.

Transcriptomic results were verified by performing RT-qPCR analyses. In particular, we confirmed the downregulation of FXN mRNA in KIKO mice and found a significant downregulation of the expression of other mitochondrial genes, such as Tfam, Mtco1, Nd1, Atp6, and Nd4 (Fig. 6a). Conversely, mRNA levels of Ucp1 remained unchanged in KIKO mice compared to WT mice. Upon cold exposure, WT mice showed up-regulation of Ucp1, Tfam, Nd1, and Atp6. In KIKO mice an increase of Ucp1, Tfam, Nd1, and Atp6 was also achieved; however, the level of up-regulation was lower than WT mice.

We also confirmed the alterations in the expression level of genes related to lipid metabolism and thermogenesis in KIKO mice. Actually, as shown in Fig. 6b, the expression of genes implicated in FA transport (i.e. Cd36, Fabp4, Cpt1b) and thermogenesis (i.e. Dio2) resulted in downregulation in KIKO mice. A slight but significant decrease of the lipase responsible for the first step of triglyceride lipolysis Atgl and its enhancer Abdh5 was also found, indicating an alteration of the triglyceride catabolism and lipid signaling. Upon cold exposure, overall these genes resulted in up-regulation both in WT and KIKO mice; however, in KIKO mice Cd36, Abdh5, Fabp4, Cpt1b, and Dio2 resulted in up-regulation to minor extent (Fig. 6b).

Through immunohistochemical analyses we confirmed the alteration of PKA lipolytic pathway in BAT of KIKO mice. Indeed, a decrease in PKA activity was occurring already at RT and a further reduction of phospho-PKA substrates was found at 4 °C with respect to WT mice (Fig. 6c). In particular, at RT the BAT of WT mice shows brown adipocytes with a diffused and stronger staining with respect to BAT of KIKO mice. Upon cold exposure, the immunostaining of phospho-PKA substrates was predominantly nuclear, in line with the notion that PKA can migrate into the nucleus during thermogenesis^32^ and phosphorylate a number of transcriptional regulators (e.g. Ppargc1a) and transcription factors triggering adipogenesis and thermogenesis as well as mitochondrial oxidative activity and biogenesis (e.g. CREB)^33,34^. Notably, the nuclear staining of phospho-PKA substrates was markedly reduced in BAT of cold-exposed KIKO mice (Fig. 6c). Moreover, in these mice we found an increase of LD-associated protein perilipin-1 (Fig. 6d), which is an inhibitor of triglyceride degradation by HSL^35^, thus highlighting the dysfunction of lipolysis in BAT of KIKO mice.

### FXN deficiency impairs thermogenesis and adipogenesis and increases susceptibility to ferroptosis in cultured adipocytes

**Fig. 7:**
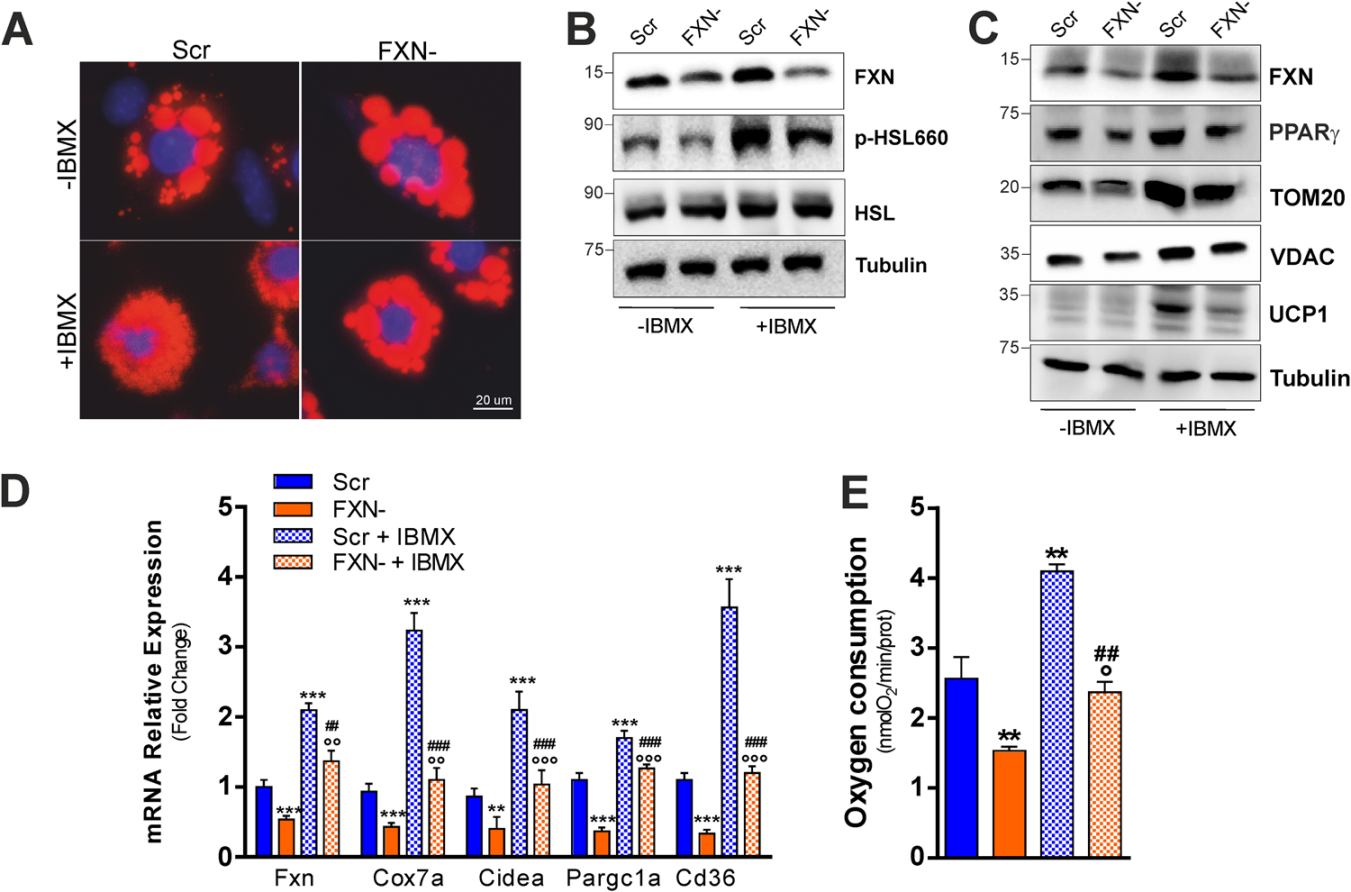
FXN deficiency impairs thermogenic program also in a brown adipocytes cell line. **a** Representative immunofluorescence analysis of LDs after staining with the neutral lipid probe Nile Red in T37i brown adipocytes transfected with a pool of siRNAs targeting FXN mRNA (FXN) or with a pool of Scr siRNAs (Scr) (*n* = 4). **b**, **c** Representative immunoblots of FXN, total and phospho-active HSL (p-HSL660) **b**, PPARγ, TOM20, VDAC, and UCP1 **c** (*n* = 4). **d** RT-qPCR analysis of genes implicated in thermogenesis (*n* = 4; **p < 0.01,***p < 0.001 vs. Scr; °°*p* < 0.01, °°°*p* < 0.001 vs. FXN-; ^##^*p* < 0.01, ^###^*p* < 0.001 vs. Scr + IBMX). **e** Cellular oxygen consumption determined through a polarographic method (*n* = 4, **p < 0.01 vs. Scr, °*p* < 0.05 vs. FXN−; ^###^*p* < 0.001 vs. Scr + IBMX).

**Fig. 8:**
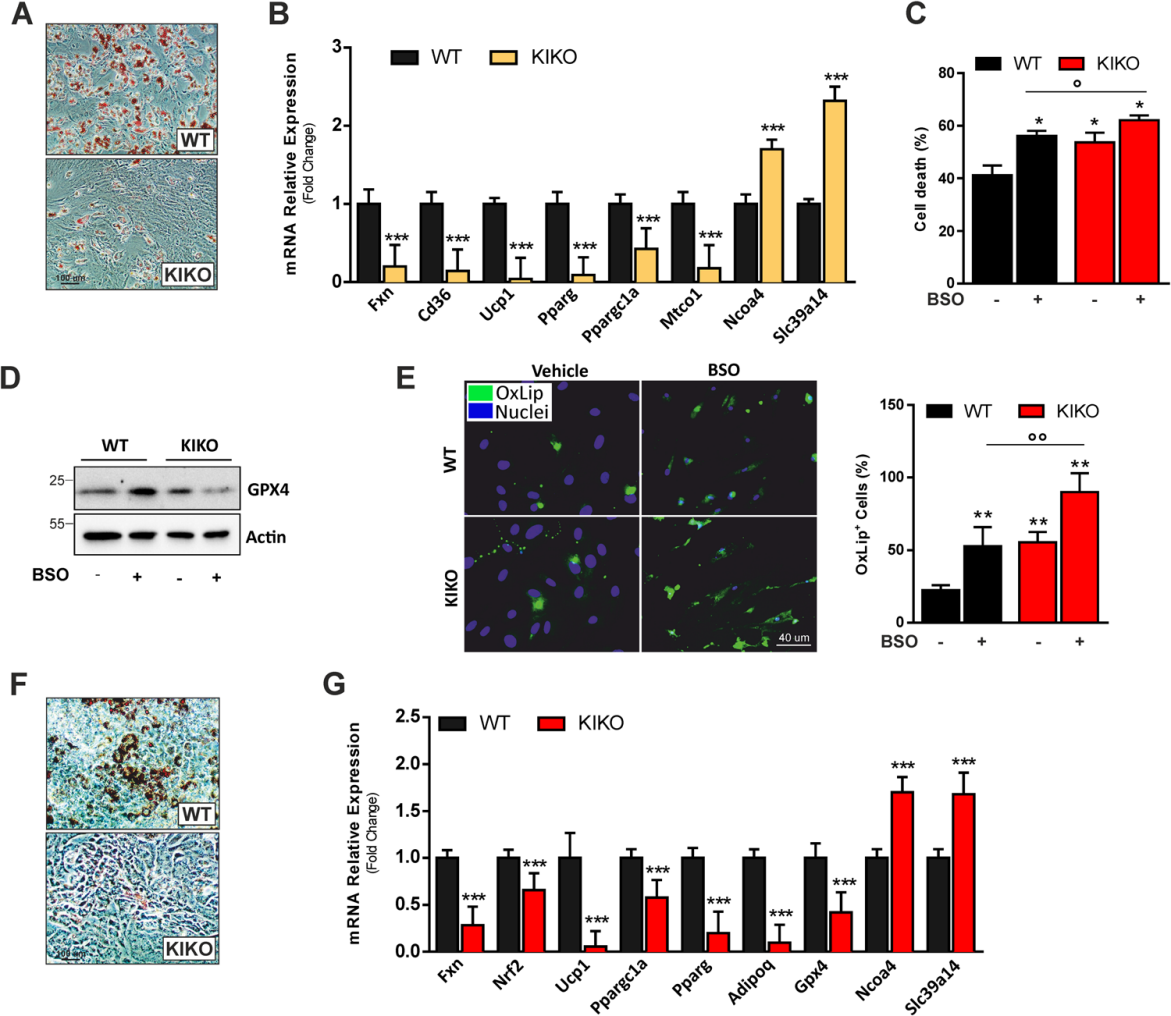
KIKO mice show affected adipogenic potential and increased ferroptosis susceptibility. **a** Representative images of stromal vascular cells (SVCs) obtained from mouse adipose depots of WT or KIKO mice, differentiated in adipocytes and stained with Oil Red O to detect LD-containing mature adipocytes (*n* = 3). **b** RT-qPCR analysis of genes implicated in adipogenesis, thermogenesis, and iron metabolism in SVCs differentiated in adipocytes (*n* = 6; ***p < 0.001 vs. WT). **c**–**e** Analysis of cell death through trypan blue staining **c**, representative immunoblots of GPX4 **d**, and lipid peroxidation (left and right panels) through C11-Bodipy staining **e** in SVCs obtained from mouse depots of WT and KIKO mice and treated with 1 mM BSO for 72 h (*n* = 3, *p < 0.05, **p < 0.01 vs. WT; °*p* < 0.05, °°*p* < 0.01 vs. BSO-treated WT). **f** Representative images of mouse embryonic fibroblasts (MEFs) obtained from WT or KIKO mouse embryos, differentiated in adipocytes and stained with Oil Red O to detect LD-containing mature adipocytes (*n* = 3). **g** RT-qPCR analysis of genes implicated in adipogenesis, thermogenesis, ferroptosis, and iron metabolism in MEFs differentiated in adipocytes (*n* = 6; ***p < 0.001 vs. WT).

To confirm that FXN deficiency affects lipid utilization and thermogenesis, we moved to an in vitro system. In cultured T37i brown adipocytes, we downregulated FXN levels through RNA interference by transfecting a pool of siRNA against FXN mRNA (FXN−). Transfection of T37i cells with a pool of scramble siRNAs was used as control (Scr). To induce thermogenic cascade, T37i were treated with isobutyl methylxanthine (IBMX), a nonspecific inhibitor of phosphodiesterase (PDE), which enhances the intracellular cAMP levels, inducing a constitutive activation of PKA. As reported in Fig. 7a, the analysis of LDs evidenced that lipolysis was likely blunted as consequence of FXN deficiency. Indeed, LDs dimension was larger in FXN− cells than Scr cells both in untreated and IBMX-treated cells. Figure 7b shows that FXN down-regulation did lead to the inhibition of lipolysis, as reduced levels of phospho-active HSL (p-HSL660), a known target of PKA activity, was elicited both upon basal condition and IBMX treatment. In line with the transcriptomic data obtained in BAT of KIKO mice, PPARγ, a thermogenic transcription factor regulating mitochondrial biogenesis and lipid catabolism, was reduced in FXN− cells under resting conditions and, in contrast to Scr cells, it remained down-regulated upon IBMX treatment (Fig. 7c). Importantly, UCP1 protein was efficiently induced by IBMX in Scr cells but not in FXN− cells. Mitochondrial proteins including VDAC and TOM20 were also reduced in FXN− cells both prior and after IBMX treatment (Fig. 7c). RT-qPCR analysis confirmed the impairment of thermogenic program, as the mRNA expression of thermogenic markers including Cox7a, Ppargc1a, Cidea, and Cd36 was significantly reduced in FXN− cells both under basal condition and upon IBMX treatment with respect to Scr cells (Fig. 7d). In line with these data, reduced oxygen consumption was also recorded in FXN− cells (Fig. 7e). Since RNAseq analyses pointed to a defective induction of adipogenic genes upon cold exposure, we then attempted at evaluating whether adipocyte differentiation potential could be impaired in KIKO mice. We hence isolated stromal vascular cells (SVCs) from subcutaneous adipose tissue, which is highly enriched with primary adipocyte precursors. Upon certain circumstances (i.e. rosiglitazone treatment), SVCs may differentiate in brown-like adipocytes displaying high mitochondrial mass and expressing detectable levels of Ucp1^36^. As reported in Fig. 8a, WT SVCs were efficiently differentiated in adipocytes as a great number of cells show adipocyte morphology with an evident accumulation of LDs. On the contrary, SVCs from KIKO mice show affected adipogenic capacity as a lower number of adipocytes was obtained. As expected, RT-qPCR analyses evidenced a marked reduction of FXN mRNA levels in KIKO with respect to WT adipocytes that was accompanied by up-regulation of mRNA levels of genes regulating iron metabolism, such as the ferritinophagy inducer Ncoa4 and the membrane iron transporter Slc39a14^37,38^ (Fig. 8b). A significant down-regulation of genes related to adipogenesis, thermogenesis and mitochondrial biogenesis including Pparγ, Ppargc1a, Ucp1, Cd36, and Mtco1 was also observed in SVCs of KIKO mice (Fig. 8b).

We then treated SVCs with buthionine sulfoximine (BSO), which is a glutathione-depleting agent able to commit ferroptotic cell death^31,39^. Under resting condition KIKO SVCs show higher percentage of cell death that was further increased compared to WT SVCs upon BSO treatment (Fig. 8c). The analysis of GPX4 protein content revealed that this antioxidant enzyme involved in protection against ferroptosis was markedly reduced in KIKO SVCs when treated with BSO (Fig. 8d). In line with these results, KIKO SVCs showed a higher amount of lipid peroxidation compared to WT SVCs both under resting conditions and upon BSO treatment (Fig. 8e). Overall these data were recapitulated in mouse embryonic fibroblasts (MEFs). In particular, KIKO MEFs were unable to undergo adipocyte differentiation, as demonstrated by the affected capacity to accumulate LDs (Fig. 8f), and to express genes related to adipogenesis, thermogenesis, and mitochondrial biogenesis (Fig. 8g). Moreover, the mRNA levels of the iron modulators Ncoa4 and Slc39a14 resulted up-regulated, whilst the mRNA levels of Gpx4 and Nrf2 resulted downregulated with respect to WT MEFs, confirming iron dysmetabolism and susceptibility to ferroptosis upon FXN deficiency.

## Discussion

Abnormal glycemic control, increased cholesterol and triglycerides levels as well as T2D are more frequent in FRDA patients than in the general population and concur in the severity of FRDA (extensively reviewed in ref. ^10^). In this study, we demonstrated that KIKO mice show several T2D-related hallmarks, including hyperlipidaemia, altered tolerance to glucose, and elevated circulating level of leptin, a peptide hormone secreted by adipose tissue whose increase is strictly related to insulin resistance and low-grade inflammatory states^40^. Overall these metabolic changes make this model suitable to decipher the events contributing to disease severity and find novel druggable targets to overcome T2D development in FRDA patients.

In the last decade, the scientific community has put a spotlight on BAT as a crucial player in the control of energy metabolism, being the main glucose and lipid clearance organ with the highest mitochondrial FA oxidation rate^41,42^. We have here reported that BAT mitochondria of KIKO mice show a significant ultrastructural alteration, lower abundance and oxygen consumption and decreased mRNA expression of electron transport chain complex subunits compared to WT mice. Upon cold exposure KIKO mice show affected cold adaptation with an overall less efficient up-regulation of canonical thermogenesis-related genes and pathways including mitochondrial and lipid metabolism genes and PPAR-signaling pathway.

It has been reported that FRDA patients show LDs accumulation in fibroblasts^17^. LDs accumulation was observed also in FXN-deficient cultured cardiomyocytes^43^ and heart of FRDA mouse models^18,19^. Hepatic steatosis in mice with a liver-specific FXN ablation^44^ and altered lipid metabolism associated with increased LDs in glial cells of the drosophila FRDA model^45^ were also observed. Down-regulation of PPARγ/PGC-1α pathway and up-regulation of lipogenic genes have been previously proposed among the mechanisms leading to LDs accumulation^17,46^. We have evidenced that FXN deficiency leads to LDs accumulation in BAT as well. Notably, the size of LDs is the result of the balance between lipid demolition and deposition. Since BAT requires plentiful FAs, which are its primary substrate, lipolysis is activated during BAT thermogenesis^33,47,48^. We have demonstrated that impaired lipid degradation could contribute to LDs accumulation, as PKA-dependent lipolysis result significantly affected both under resting condition and upon cold exposure. As a consequence, a significant reduction of the active form of HSL and increase of the LD-associated inhibitor of lipolysis perilipin was observed in BAT of KIKO mice.

Importantly, besides acting as fuels to sustain the electron transport chain flow, FAs are involved in the regulation of UCP1, or directly activate UCP1-mediated energy dissipation and heat production^33^. UCP1 was not found altered under resting condition in KIKO mice. However, the impaired lipid degradation observed in KIKO mice likely limits the funneling of FAs into mitochondria with consequent affected capacity to produce heat. Accordingly, β-oxidation was not disclosed among the enriched pathways in BAT of cold-exposed KIKO mice and a diminished expression of the mitochondrial FA carrier Cpt1b was found. Notably, intracellular purine nucleotides exert an inhibitory action on UCP1 protein^49^. It was recently demonstrated that upon thermogenic stimuli, brown adipocyte expression of enzymes implicated in purine metabolic remodeling is altered^49^. In particular, an overexpression of genes implicated in purine nucleotide degradation was found that was associated with a down-regulation of genes involved in purine nucleotide synthesis. In BAT of KIKO mice exposed to cold we found an overrepresentation of genes pertaining purine nucleotide biosynthetic process. Therefore, these data collectively indicate that UCP1, in addition to being up-regulated to lesser extent than WT mice upon cold exposure, is less efficient in impinging heat production.

To exclude that the alterations found in BAT of KIKO mice could be the result of systemic adaptive responses to FXN deficiency, we validated our findings in cultured brown adipocytes down-regulating FXN. By igniting thermogenic program via the PKA agonist IBMX we confirmed that FXN deficiency leads to the inhibition of lipolysis and affected up-regulation of genes related to the thermogenic cascade.

Another process having a critical role in maintaining thermogenic efficiency of BAT is the differentiation of resident brown fat adipocytes de novo that assures BAT regeneration and avoids its loss^50^. Transcriptomic data evidenced brown fat cell differentiation and adipogenesis as biological process, and pathway affected upon cold exposure in BAT of our mouse model. We confirmed such hypothesis by analyzing the adipogenic potential of SVCs isolated from adipose depots that result unable to fully differentiate in thermogenic adipocytes in KIKO mice.

It is important to notice that a less proficient up-regulation of p53 signaling pathway was observed in BAT of KIKO mice upon cold exposure. p53 is a stress-responsive transcription factor that was reported to exert a positive regulatory effect on brown adipocyte differentiation^51^ and BAT thermogenesis^52^. This evidence corroborates our idea that BAT dysfunction could at least in part depend on altered capacity of FXN-deficient adipocyte precursors to efficiently differentiate, and express mitochondrial and thermogenic genes.

Ferroptosis occurrence could be another cause of BAT dysfunction. Transcriptomic results indicated that upon cold stress condition, the KEGG pathway of ferroptosis was significantly up-regulated in KIKO mice along with genes related to ROS metabolism. Along this line, we found a decrease of NRF2 and GPX4, altered expression of genes controlling iron metabolism and increased susceptibility to ferroptosis in brown adipocyte precursors, as well as in mature adipocytes isolated from KIKO mice. These results are in accordance with the findings pointing to oxidative stress, iron overload, and ferroptosis among the main pathogenic factors in FRDA^6,27,53–55^. Interestingly, NRF2, besides being an up-stream modulator of the expression of antioxidant genes and protection against oxidative stress/ferroptosis^56,57^, positively regulates enzymes involved in mitochondrial oxidation of FAs^29^ and adipogenesis^58,59^. Based on this, NRF2 inducers, already proposed as FRDA therapeutics^53,54,60^ could be also advantageous for preserving BAT integrity and mitigating metabolic disturbances of FRDA patients via a BAT-dependent manner. Accordingly, NRF2 targeting has been proposed as promising for treating T2D^61^.

BAT studies carried out in human fetuses and infants indicate that the tissue is widely distributed during these developmental stages and that the thermogenic capacity of BAT develops with gestational age reaching its maximum in infancy and early childhood when the demands for thermogenesis can be expected to be especially high^62^. Several studies suggest that BAT dysfunction during gestation and early childhood negatively influences metabolic health predisposing to T2D development later in life^63–65^. Therefore, it is possible to postulate that FRDA patients would have experienced BAT dysfunction early in life leading to disruption of systemic metabolic homeostasis. Subcutaneous white adipose tissue also participates in the maintenance of body metabolic homeostasis and responds to cold by activating a thermogenic response^66^. Whether an impairment of this adipose depot is also operative in KIKO mice and contributes to metabolic complications in FRDA is currently under investigation in our laboratory.

In conclusion, by deeply characterizing KIKO mice at metabolic level we have provided multiple lines of evidence that FXN deficiency in mice leads to clinical-pathological features parallel to those observed in diabetic patients. Among the metabolic parameters we have evidenced that the lipolytic and thermogenic activities of BAT are reduced, thus providing the possibilities of targeting BAT that might result in therapeutic benefits in FRDA.

## Materials and methods

### Animals

Mouse experimentation was conducted in accordance with accepted standard of humane animal care after the approval by relevant local (Institutional Animal Care and Use Committee, Tor Vergata University) and national (Ministry of Health, license no. 324/2018-PR) committees. Unless otherwise stated, mice were maintained at 21.0± °C and 55.0 ± 5.0% relative humidity under a 12 h/12 h light/dark cycle (lights on at 6:00 a.m., lights off at 6:00 p.m.). Food and water were given ad libitum. Experiments were carried out according to institutional safety procedures.

Knock-in knock-out (KIKO) mice were purchased from Jackson Laboratories (#012329). Littermate C57BL/6 mice (WT) were used as controls. Researchers were blinded to genotypes at the time of testing. Only WT and KIKO male mice were used and divided in the following groups: (1) 6-month-old WT and KIKO mice (*n* = 12 each group); (2) 8-month-old WT and KIKO mice (*n* = 12 each group);

For cold exposure experiments additional male WT and KIKO at 6 months of age were divided in the following groups: (1) WT and KIKO mice maintained at room temperature (*n* = 6 each group); (2) WT and KIKO mice maintained at 4 °C for 12 h (*n* = 6 each group). Rectal temperature was measured by high precision (±0.1 °C) rectal probe for mice (RET-3, ThermoWorks, Alpine, UT, USA).

### Bio-clinical and adipokine analyses

Prior to bio-clinical analyses, mice were starved for 2 h. After blood collection, bio-clinical analyses were performed by colorimetric methods. In particular, glucose, cholesterol, triglycerides, creatinine, total plasma proteins, albumin, and urea were measured through the automatized KeyLab analyzer (BPCBioSed, Italy) using specific assay kits (BPCBioSed).

Serum adiponectin, FGF21, and leptin levels were measured through a Mouse Magnetic Luminex Screening Assay (R&D System, Minneapolis, MN, USA).

For the glucose tolerance test (OGTT), mice were subjected to fasting for 12 h, followed by oral gavage with 2 g of dextrose/kg body mass. At the indicated time points, blood was collected from the tail vein and glycaemia measured using a glucometer (Bayer Countur XT, Bayer Leverkusen, Germany).

### Indirect calorimetry

Indirect calorimetry performed using LabMaster (TSE Systems, Bad Homburg, Germany) as previously described^67^. Oxygen consumption (VO2), carbon dioxide production (VCO_2_), and REE were recorded every 15 min for 24 h, and the data were averaged for each mouse.

### Histochemical analysis

Formalin-fixed paraffin-embedded (BAT explants were cut into 3 μm sections and stained with hematoxylin and eosin (H&E) prior to microscope analysis). LD diameters were measured in three fields (300 droplets total at least) with ImageJ. For correlative purpose, an average score was derived for each sample. Perilipin and phospho-PKA substrate levels were investigated by immunohistochemistry on tissue sections. After antigen retrieval with citrate buffer (pH 6.0), sections were incubated at room temperature with the following primary antibodies:rabbit polyclonal antibody anti-perilipin diluted 1:1000 (9349T, Cell Signalling Technology) and rabbit polyclonal antibody anti-phospho-PKA substrates diluted 1:100 (9621S, Cell Signalling Technology). Negative controls were obtained by omitting primary antibodies. Immunohistochemical reactions were visualized by DAB as the chromogen from MACH 1 Universal HRP-Polymer Detection (Biocare Medical, Concord, MA, USA).

### Ultrastructural analysis

BAT samples from animal models were fixed in 2.5% glutaraldehyde in 0.1 M cacodylate buffer for the morphological study. After fixation, dehydration, and impregnation, samples were included in epoxy resins and acrylic, cut at the ultramicrotome and processed for the ultrastructural study of brown adipocytes by electron microscopy. In particular, morphological aspects of the mitochondria, such as the presence and density of cristae, were evaluated. Ultrastructural images were collected with a transmission electron microscope FEI TecnaiTM (Hillsboro, OR, USA), equipped with a dedicated Imaging Software System.

### Cells and treatments

T37i cell line was gently provided by Prof. Marc Lombes (Inserm U693, Paris, France), cultured and differentiated as described by Nakae et al.^68^. Differentiated T37i brown adipocytes were transfected with a pool of three target-specific siRNAs against FXN mRNA FXN or scramble siRNAs (Santa Cruz Biotechnology, Dallas, TX, USA) by using DeliverX Plus kit (Affymetrix, Santa Clara, CA, USA). Treatment with isobutyl methyl xanthine (IBMX) was carried out at concentration of 0.5 mM for 4 h. For LD detection, cells were stained with Nile Red (0.25 μg/ml,10 min). Staining with Hoechst 33342 (1 µg/ml, 10 min) was used to counterstain nuclei. Images were visualized by Nikon Eclipse TE200 epifluorescence microscope (Nikon, Florence, Italy) connected to a CCD camera. Images were captured under constant exposure time, gain and offset.

SVCs were isolated from subcutaneous adipose tissue and differentiated in brown-like adipocytes according to Aune et al.^36^. Oil Red O was used to detect intracellular triglycerides content as previously described^67^. MEFs were prepared from embryos at E13.5 as previously described^69^. MEFs were cultured in DMEM with 10% FBS and used for experiments from the 2nd to the 3rd passage in culture.

### Mitochondrial oxygen consumption and lipid peroxidation assay

Oxygen consumption was determined in cells and crude mitochondria and by using the Oxygraph Plus oxygen electrode system (Hansatech Instruments Ltd., Norfolk, UK). Intact cells were resuspended (1 × 10^6^/ml) in culture medium without FBS. Crude mitochondria were isolated from BAT as previously described^70^ and resuspended in an appropriate mitochondrial activity buffer (70 mM sucrose, 220 mM mannitol, 2 mM HEPES buffer, 5 mM magnesium chloride, 5 mM potassium phosphate, 1 mM EDTA, 5 mM succinic acid, and 0.1% fatty acid free bovine serum albumin, pH 7.4). Oxygen consumption rate was recorded at 37 °C for 10 min and normalized for protein concentration.

Cells were incubated with C11-BODIDY 581/591 (Lipid Peroxidation Sensor, Invitrogen) at final concentration of 10 μM for 30 min at 37 °C and fixed with 10% formalin solution. Lipid peroxidation was analyzed through fluorescent microscopy by counting the percentage of positive cells in eight different fields.

### RNA-seq data expression quantification and functional enrichment analysis

The adipose tissue samples were subject to RNA-sequencing using an Illumina NextSeq500 and the indexed libraries were prepared from 1 μg-purified RNA with TruSeq-stranded mRNA (Illumina) Library Prep Kit according to the manufacturer’s instructions. The quality of the single-end reads was evaluated with *FastQC* v.0.11.5 (https://www.bioinformatics.babraham.ac.uk/projects/fastqc/). All the *fastqc* files were filtered to remove low-quality reads and adapters with *Trimmomatic* v.0.36^71^. The resulting reads were mapped to the *Mus musculus* genome (GRCm38) with *HISAT2* v.2.1.0^72^ using default parameters, while *Stringtie* v1.3.4d^73^ was applied to the BAM files obtained with *HISAT2* to generate expression estimates and to quantify the transcript abundance as transcripts per kilobase per million of mapped reads (TPM). The count matrices generated by *Stringtie* were imported in *R*, where differential expression analysis was performed using the *Deseq2* package^74^ to compare the two different conditions. The functional annotation was performed through the *AnnotationDbi* R library (http://bioconductor.org/packages/release/bioc/html/AnnotationDbi.html).

Differential expressed genes were selected with threshold of Log_2_FC > 0.58 (*p* < 0.05). Functional enrichment analysis including GO and Kyoto Encyclopedia of Genes and Genomes (KEGG) pathway was performed by using the ClueGo plugin of the Cytoscape v3.7.1. ClueGo settings were: enrichment only, Benjamini–Hochberg false discovery rate (FDR) correction, GO Term Restriction Level 3–8 and 3 genes/4% minimum, GO Term Connection (Kappa) minimum 0.4, GO Term Grouping was on, with an initial group size of 3 and Group Merging set at 50%. Only pathway with pV < 0.05 were shown. Funrich v3.0 tool (http://funrich.org/index.html) with default settings was alternatively used for functional enrichment analyses.

### Real-time PCR

Total RNA was extracted using TRI Reagent® (Sigma-Aldrich). RNA (3 μg) was retro-transcripted by using M-MLV (Promega, Madison, WI). qPCR was performed in triplicate by using validated qPCR primers (BLAST), Applied Biosystems™ *Power*™ SYBR™ Green Master Mix, and the QuantStudio3 Real-Time PCR System (Thermo Fisher, Whaltam, MA, USA) as previously described^75^. mRNA levels were normalized to actin mRNA, and the relative mRNA levels were determined through the 2^−ΔΔCt^ method.

### Immunoblotting

Tissues or cells were lysed in RIPA buffer (50 mM Tris–HCl, pH 8.0, 150 mM NaCl, 12 mM deoxycholic acid, 0.5% Nonidet P-40, and protease and phosphatase inhibitors). Nuclear and cytoplasmic fractions were extracted from BAT explants as previously described^76^. Five micrograms of proteins were loaded on SDS–PAGE and subjected to Western blotting. Nitrocellulose membranes were incubated with anti-HSL (4107, Cell Signalling Technology), anti-p-HSL660 (4126T, Cell Signalling Technology), anti-p-HSL565 (4137P, Cell Signalling Technology), anti-UCP1 (CS-14670S, Cell Signalling Technology) anti-p-PKA substrates (9621S, Cell Signalling Technology), anti-FXN (sc-25820, Santa Cruz Biotechnology), anti-NRF2 (sc-722, Santa Cruz Biotechnology), anti-PPARγ (sc-7196, Santa Cruz Biotechnology), anti-Tomm20 (sc-11415, Santa Cruz Biotechnology), anti-Vdac1 (sc-8828, Santa Cruz Biotechnology), anti-Tubulin (T9026, Sigma-Aldrich) primary antibodies at 1:1000 dilution. Successively, membranes were incubated with the appropriate horseradish peroxidase-conjugated secondary antibodies. Immunoreactive bands were detected by a FluorChem FC3 System (Protein-Simple, San Jose, CA, USA) after incubation of the membranes with ECL Selected Western Blotting Detection Reagent (GE Healthcare, Pittsburgh, PA, USA). Densitometric analyses of the immunoreactive bands were performed by the FluorChem FC3 Analysis Software.

### Statistical analysis

The results are presented as means ± S.D. Statistical analyses were carried out by using the Student’s *t* test to compare the means of two groups. One-way ANOVA followed by Tukey’s test was used for comparing the means of more than two groups. Differences were considered to be significant at *p* < 0.05.

## References

[1] Abrahao A (2015). Milestones in Friedreich ataxia: more than a century and still learning. Neurogenetics.

[2] Maio N, Rouault TA (2015). Iron-sulfur cluster biogenesis in mammalian cells: new insights into the molecular mechanisms of cluster delivery. Biochim. Biophys. Acta.

[3] Koeppen AH (2015). The pathogenesis of cardiomyopathy in *Friedreich ataxia*. PLoS ONE.

[4] Koeppen AH, Mazurkiewicz JE (2013). Friedreich ataxia: neuropathology revised. J. Neuropathol. Exp. Neurol..

[5] Koeppen AH, Davis AN, Morral JA (2011). The cerebellar component of Friedreich’s ataxia. Acta Neuropathol..

[6] Cotticelli MG (2019). Ferroptosis as a Novel Therapeutic Target for Friedreich’s ataxia. J. Pharm. Exp. Ther..

[7] Cnop M, Mulder H, Igoillo-Esteve M (2013). Diabetes in Friedreich ataxia. J. Neurochem..

[8] Isaacs CJ (2016). Effects of genetic severity on glucose homeostasis in Friedreich ataxia. Muscle Nerve.

[9] Raman SV (2011). Impaired myocardial perfusion reserve and fibrosis in Friedreich ataxia: a mitochondrial cardiomyopathy with metabolic syndrome. Eur. Heart J..

[10] Tamarit J, Obis E, Ros J (2016). Oxidative stress and altered lipid metabolism in Friedreich ataxia. Free Radic. Biol. Med.

[11] Cnop M (2012). Central role and mechanisms of beta-cell dysfunction and death in friedreich ataxia-associated diabetes. Ann. Neurol..

[12] Chondronikola M (2014). Brown adipose tissue improves whole-body glucose homeostasis and insulin sensitivity in humans. Diabetes.

[13] Chondronikola M (2016). Brown adipose tissue activation is linked to distinct systemic effects on lipid metabolism in humans. Cell Metab..

[14] Hankir MK, Klingenspor M (2018). Brown adipocyte glucose metabolism: a heated subject. EMBO Rep..

[15] Sidossis L, Kajimura S (2015). Brown and beige fat in humans: thermogenic adipocytes that control energy and glucose homeostasis. J. Clin. Invest..

[16] Blondin DP (2017). Inhibition of intracellular triglyceride lipolysis suppresses cold-induced brown adipose tissue metabolism and increases shivering in humans. Cell Metab..

[17] Coppola G (2009). Functional genomic analysis of frataxin deficiency reveals tissue-specific alterations and identifies the PPARgamma pathway as a therapeutic target in Friedreich’s ataxia. Hum. Mol. Genet..

[18] Stram AR (2017). Progressive mitochondrial protein lysine acetylation and heart failure in a model of Friedreich’s ataxia cardiomyopathy. PLoS ONE.

[19] Puccio H (2001). Mouse models for Friedreich ataxia exhibit cardiomyopathy, sensory nerve defect and Fe–S enzyme deficiency followed by intramitochondrial iron deposits. Nat. Genet..

[20] Sacks H, Symonds ME (2013). Anatomical locations of human brown adipose tissue: functional relevance and implications in obesity and type 2 diabetes. Diabetes.

[21] Flachs P, Rossmeisl M, Kuda O, Kopecky J (2013). Stimulation of mitochondrial oxidative capacity in white fat independent of UCP1: a key to lean phenotype. Biochim. Biophys. Acta.

[22] McMackin MZ, Henderson CK, Cortopassi GA (2017). Neurobehavioral deficits in the KIKO mouse model of Friedreich’s ataxia. Behav. Brain Res.

[23] Li Y, Ding L, Hassan W, Abdelkader D, Shang J (2013). Adipokines and hepatic insulin resistance. J. Diabetes Res..

[24] Adams-Huet B, Devaraj S, Siegel D, Jialal I (2014). Increased adipose tissue insulin resistance in metabolic syndrome: relationship to circulating adipokines. Metab. Syndr. Relat. Disord..

[25] Finucane FM (2009). Correlation of the leptin:adiponectin ratio with measures of insulin resistance in non-diabetic individuals. Diabetologia.

[26] Sahdeo S (2014). Dyclonine rescues frataxin deficiency in animal models and buccal cells of patients with Friedreich’s ataxia. Hum. Mol. Genet..

[27] Shan Y (2013). Frataxin deficiency leads to defects in expression of antioxidants and Nrf2 expression in dorsal root ganglia of the Friedreich’s ataxia YG8R mouse model. Antioxid. Redox Signal.

[28] Xie Y (2016). Ferroptosis: process and function. Cell Death Differ..

[29] Esteras N, Dinkova-Kostova AT, Abramov AY (2016). Nrf2 activation in the treatment of neurodegenerative diseases: a focus on its role in mitochondrial bioenergetics and function. Biol. Chem..

[30] Dixon SJ (2012). Ferroptosis: an iron-dependent form of nonapoptotic cell death. Cell.

[31] Stockwell BR (2017). Ferroptosis: a regulated cell death nexus linking metabolism, redox biology, and disease. Cell.

[32] Rim JS, Xue B, Gawronska-Kozak B, Kozak LP (2004). Sequestration of thermogenic transcription factors in the cytoplasm during development of brown adipose tissue. J. Biol. Chem..

[33] Cannon B, Nedergaard J (2004). Brown adipose tissue: function and physiological significance. Physiol. Rev..

[34] Baldelli S, Lettieri Barbato D, Tatulli G, Aquilano K, Ciriolo MR (2014). The role of nNOS and PGC-1alpha in skeletal muscle cells. J. Cell Sci..

[35] Grahn TH (2013). FSP27 and PLIN1 interaction promotes the formation of large lipid droplets in human adipocytes. Biochem. Biophys. Res. Commun..

[36] Aune UL, Ruiz L, Kajimura S (2013). Isolation and differentiation of stromal vascular cells to beige/brite cells. J. Vis. Exp..

[37] Nam H (2013). ZIP14 and DMT1 in the liver, pancreas, and heart are differentially regulated by iron deficiency and overload: implications for tissue iron uptake in iron-related disorders. Haematologica.

[38] Dowdle WE (2014). Selective VPS34 inhibitor blocks autophagy and uncovers a role for NCOA4 in ferritin degradation and iron homeostasis in vivo. Nat. Cell Biol..

[39] Sun Y, Zheng Y, Wang C, Liu Y (2018). Glutathione depletion induces ferroptosis, autophagy, and premature cell senescence in retinal pigment epithelial cells. Cell Death Dis..

[40] Francisco V (2018). Obesity, fat mass and immune system: role for leptin. Front. Physiol..

[41] Bartelt A (2011). Brown adipose tissue activity controls triglyceride clearance. Nat. Med..

[42] Doh KO (2005). Interrelation between long-chain fatty acid oxidation rate and carnitine palmitoyltransferase 1 activity with different isoforms in rat tissues. Life Sci..

[43] Obis E, Irazusta V, Sanchis D, Ros J, Tamarit J (2014). Frataxin deficiency in neonatal rat ventricular myocytes targets mitochondria and lipid metabolism. Free Radic. Biol. Med..

[44] Martelli A (2012). Clinical data and characterization of the liver conditional mouse model exclude neoplasia as a non-neurological manifestation associated with Friedreich’s ataxia. Dis. Model Mech..

[45] Navarro JA (2010). Altered lipid metabolism in a Drosophila model of Friedreich’s ataxia. Hum. Mol. Genet..

[46] Sutak R (2008). Proteomic analysis of hearts from frataxin knockout mice: marked rearrangement of energy metabolism, a response to cellular stress and altered expression of proteins involved in cell structure, motility and metabolism. Proteomics.

[47] Nedergaard J, Bengtsson T, Cannon B (2011). New powers of brown fat: fighting the metabolic syndrome. Cell Metab..

[48] Townsend KL, Tseng YH (2014). Brown fat fuel utilization and thermogenesis. Trends Endocrinol. Metab..

[49] Fromme T (2018). Degradation of brown adipocyte purine nucleotides regulates uncoupling protein 1 activity. Mol. Metab..

[50] Birerdinc A, Jarrar M, Stotish T, Randhawa M, Baranova A (2013). Manipulating molecular switches in brown adipocytes and their precursors: a therapeutic potential. Prog. Lipid Res..

[51] Al-Massadi O (2016). Pharmacological and genetic manipulation of p53 in brown fat at adult but not embryonic stages regulates thermogenesis and body weight in male mice. Endocrinology.

[52] Molchadsky A (2013). p53 is required for brown adipogenic differentiation and has a protective role against diet-induced obesity. Cell Death Differ..

[53] Abeti R, Baccaro A, Esteras N, Giunti P (2018). Novel Nrf2-Inducer prevents mitochondrial defects and oxidative stress in Friedreich’s ataxia models. Front. Cell Neurosci..

[54] Petrillo Sara, Piermarini Emanuela, Pastore Anna, Vasco Gessica, Schirinzi Tommaso, Carrozzo Rosalba, Bertini Enrico, Piemonte Fiorella (2017). Nrf2-Inducers Counteract Neurodegeneration in Frataxin-Silenced Motor Neurons: Disclosing New Therapeutic Targets for Friedreich’s Ataxia. International Journal of Molecular Sciences.

[55] La Rosa P (2019). Nrf2 induction re-establishes a proper neuronal differentiation program in Friedreich’s ataxia neural stem cells. Front. Cell. Neurosci..

[56] Dodson, M., Castro-Portuguez, R. & Zhang, D. D. NRF2 plays a critical role in mitigating lipid peroxidation and ferroptosis. *Redox. Biol*. **23**, 101107 (2019). 10.1016/j.redox.2019.101107 PMC6859567 30692038

[57] Faraonio R (2006). Transcription regulation in NIH3T3 cell clones resistant to diethylmaleate-induced oxidative stress and apoptosis. Antioxid. Redox Signal..

[58] Pi J (2010). Deficiency in the nuclear factor E2-related factor-2 transcription factor results in impaired adipogenesis and protects against diet-induced obesity. J. Biol. Chem..

[59] Hou Y (2012). Nuclear factor erythroid-derived factor 2-related factor 2 regulates transcription of CCAAT/enhancer-binding protein beta during adipogenesis. Free Radic. Biol. Med..

[60] Petrillo, S., D’Amico, J., La Rosa, P., Bertini, E. S. & Piemonte, F. Targeting NRF2 for the treatment of Friedreich’s ataxia: a comparison among drugs. *Int. J. Mol. Sci*. **20**, pii E5211 (2019). 10.3390/ijms20205211 PMC6829337 31640150

[61] David JA, Rifkin WJ, Rabbani PS, Ceradini DJ (2017). The Nrf2/Keap1/ARE pathway and oxidative stress as a therapeutic target in Type II diabetes mellitus. J. Diabetes Res..

[62] Lidell ME (2019). Brown adipose tissue in human infants. Handb. Exp. Pharm..

[63] Entringer S (2017). Association between supraclavicular brown adipose tissue composition at birth and adiposity gain from birth to 6 months of age. Pediatr. Res..

[64] Liang X (2016). Maternal high-fat diet during lactation impairs thermogenic function of brown adipose tissue in offspring mice. Sci. Rep..

[65] Lettieri-Barbato D (2017). Maternal high calorie diet induces mitochondrial dysfunction and senescence phenotype in subcutaneous fat of newborn mice. Oncotarget.

[66] Herz CT, Kiefer FW (2019). Adipose tissue browning in mice and humans. J. Endocrinol..

[67] Lettieri-Barbato D, Cannata SM, Casagrande V, Ciriolo MR, Aquilano K (2018). Time-controlled fasting prevents aging-like mitochondrial changes induced by persistent dietary fat overload in skeletal muscle. PLoS ONE.

[68] Nakae J (2008). Forkhead transcription factor FoxO1 in adipose tissue regulates energy storage and expenditure. Diabetes.

[69] Durkin, M. E., Qian, X., Popescu, N. C. & Lowy, D. R. Isolation of mouse embryo fibroblasts. *Bio Protoc.* **3**, e908 (2013). 10.21769/bioprotoc.908 PMC4928858 27376106

[70] Lettieri Barbato D (2015). Dietary fat overload reprograms brown fat mitochondria. Front. Physiol..

[71] Bolger AM, Lohse M, Usadel B (2014). Trimmomatic: a flexible trimmer for Illumina sequence data. Bioinformatics.

[72] Kim D, Langmead B, Salzberg SL (2015). HISAT: a fast spliced aligner with low memory requirements. Nat. Methods.

[73] Pertea M (2015). StringTie enables improved reconstruction of a transcriptome from RNA-seq reads. Nat. Biotechnol..

[74] Love MI, Huber W, Anders S (2014). Moderated estimation of fold change and dispersion for RNA-seq data with DESeq2. Genome Biol..

[75] Aquilano K (2016). Adipose triglyceride lipase decrement affects skeletal muscle homeostasis during aging through FAs-PPARalpha-PGC-1alpha antioxidant response. Oncotarget.

[76] Lettieri Barbato D, Tatulli G, Aquilano K, Ciriolo MR (2015). Mitochondrial hormesis links nutrient restriction to improved metabolism in fat cell. Aging.

