# Correction: Frataxin deficiency induces lipid accumulation and affects thermogenesis in brown adipose tissue

**DOI:** 10.1038/s41419-020-2347-x

**Published:** 2020-03-03

**Authors:** Riccardo Turchi, Flavia Tortolici, Giulio Guidobaldi, Federico Iacovelli, Mattia Falconi, Stefano Rufini, Raffaella Faraonio, Viviana Casagrande, Massimo Federici, Lorenzo De Angelis, Simone Carotti, Maria Francesconi, Maria Zingariello, Sergio Morini, Roberta Bernardini, Maurizio Mattei, Piergiorgio La Rosa, Fiorella Piemonte, Daniele Lettieri-Barbato, Katia Aquilano

**Affiliations:** 1 Department Biology, University of Rome Tor Vergata, via della Ricerca Scientifica, Rome, Italy; 2 Department of Molecular Medicine and Medical Biotechnologies, University of Naples Federico II, Naples, Italy; 3 Department of Systems Medicine, University of Rome Tor Vergata, Rome, Italy; 4 Unit of Microscopic and Ultrastructural Anatomy, University Campus Bio-Medico, Rome, Italy; 5 Interdepartmental Service Center-Station for Animal Technology (STA), University of Rome Tor Vergata, Rome, Italy; 6 Unit of Neuromuscular and Neurodegenerative Diseases, IRCCS Bambino Gesù Children’s Hospital, Rome, Italy; 7 IRCCS Fondazione Santa Lucia, 00143 Rome, Italy

**Keywords:** Biological sciences, Biochemistry

**Correction to**: **Cell Death and Disease**

10.1038/s41419-020-2253-2 published online 23 January 2020

Since online publication of this article, the authors noticed that there was a basic citation error in PubMed citation data. Specifically, the name of the author “Piergiorgio La Rosa” is cited as "Rosa P” in the PubMed citation, when it should be “La Rosa P”, “La Rosa” being the surname and “Piergiorgio” the name of the author.

The HTML version of the article has been modified accordingly.

